# Glioma stem cells are more aggressive in recurrent tumors with malignant progression than in the primary tumor, and both can be maintained long-term *in vitro*

**DOI:** 10.1186/1471-2407-8-304

**Published:** 2008-10-22

**Authors:** Qiang Huang, Quan-Bin Zhang, Jun Dong, Yin-Yan Wu, Yun-Tian Shen, Yao-Dong Zhao, Yu-De Zhu, Yi Diao, Ai-Dong Wang, Qing Lan

**Affiliations:** 1 Department of Neurosurgery and Brain Tumor Research Laboratory, the Second Affiliated Hospital of Suzhou University, Suzhou 215004, PR China; 2 Department of Neurosurgery, Kowloon Hospital, Shanghai Jiaotong University Medical School, Suzhou 215021, PR China; 3 Departments of Radiation Oncology, the Second Affiliated Hospital of Suzhou University, Suzhou 215004, PR China

## Abstract

**Background:**

Despite the advances made during decades of research, the mechanisms by which glioma is initiated and established remain elusive. The discovery of glioma stem cells (GSCs) may help to elucidate the processes of gliomagenesis with respect to their phenotype, differentiation and tumorigenic capacity during initiation and progression. Research on GSCs is still in its infancy, so no definitive conclusions about their role can yet be drawn. To understand the biology of GSCs fully, it is highly desirable to establish permanent and biologically stable GSC lines.

**Methods:**

In the current study, GSCs were isolated from surgical specimens of primary and recurrent glioma in a patient whose malignancy had progressed during the previous six months. The GSCs were cryopreserved and resuscitated periodically during long-term maintenance to establish glioma stem/progenitor cell (GSPC) lines, which were characterized by immunofluorescence, flow cytometry and transmission electronic microscopy. The primary and recurrent GSPC lines were also compared in terms of in vivo tumorigenicity and invasiveness. Molecular genetic differences between the two lines were identified by array-based comparative genomic hybridization and further validated by real-time PCR.

**Results:**

Two GSPC lines, SU-1 (primary) and SU-2 (recurrent), were maintained *in vitro* for more than 44 months and 38 months respectively. Generally, the potentials for proliferation, self-renewal and multi-differentiation remained relatively stable even after a prolonged series of alternating episodes of cryopreservation and resuscitation. Intracranial transplantation of SU-1 cells produced relatively less invasive tumor mass in athymic nude mice, while SU-2 cells led to much more diffuse and aggressive lesions strikingly recapitulated their original tumors. Neither SU-1 nor SU-2 cells reached the terminal differentiation stage under conditions that would induce terminal differentiation in neural stem cells. The differentiation of most of the tumor cells seemed to be blocked at the progenitor cell phase: most of them expressed nestin but only a few co-expressed differentiation markers. Transmission electron microscopy showed that GSCs were at a primitive stage of differentiation with low autophagic activity. Array-based comparative genomic hybridization revealed genetic alterations common to both SU-1 and SU-2, including amplification of the oncogene *EGFR *and deletion of the tumor suppressor *PTEN*, while some genetic alterations such as amplification of *MTA1 *(metastasis associated gene 1) only occurred in SU-2.

**Conclusion:**

The GSPC lines SU-1 and SU-2 faithfully retained the characteristics of their original tumors and provide a reliable resource for investigating the mechanisms of formation and recurrence of human gliomas with progressive malignancy. Such investigations may eventually have major impacts on the understanding and treatment of gliomas.

## Background

Recent decades have seen only limited progress in treatment trials and basic research on human glioma, the most common central nervous malignancy. This is partly because previously-established glioma cell lines are composed of morphologically and functionally diverse cells that express a variety of neural lineage markers [1]. It is now generally accepted that these previously-established serum-based cell lines do not replicate the major biological features, particularly the stem cells, of human cancers. Therefore, there is an urgent need for new and clinically relevant *in vitro* model systems for studying tumor biology and conducting preclinical screening of drugs for malignant brain tumors.

There is overwhelming evidence that glioma tissue contains stem cells that are broadly similar to neural stem cells but differ from them in important ways [2-5]. Although CD133, a 120 kDa cell-surface marker of normal human neural stem cells (NSCs), is not a specific marker or gold standard for identifying glioma stem cells, it has been used in most relevant studies for enriching tumor stem-like cells from brain tumors. Vescovi offered a functional definition of brain tumor stem cells, namely: brain tumor cells should qualify as stem cells if they show cancer-initiating ability upon orthotopic implantation, extensive self-renewal ability demonstrated either ex vivo or in vivo, karyotypic or genetic alterations, aberrant differentiation properties, capacity to generate non-tumorigenic end cells, and multilineage differentiation capacity [6]. Because this subpopulation of glioma cells, generally called glioma stem cells (GSCs), may play an extremely critical role in the initiation and recurrence of gliomas, studies focusing on GSCs have been promoted rapidly. However, conclusions about the biological features of GSCs are not always consistent and sometimes even confusing [1,7-14]. Most investigators believe that short-term cultured stem cells are superior to those maintained long-term. However, since glioma tissues are in very short supply, it is difficult to find tumor stem cells readily for either biological or preclinical drug studies. Therefore, permanent GSC lines could serve such purposes better than GSCs maintained short-term.

Tumor recurrence is the primary cause of treatment failure and death in glioma patients. Although cancer stem cells are now widely believed to be responsible for tumor recurrence, we do not know whether such cells are exactly the same in recurrent tumors as in the primary tumor in cases of malignancy progression.

We have isolated GSCs from surgical specimens of both primary and recurrent gliomas in recent years. Fortunately, GSCs from primary and recurrent tumors in the same patient were screened out and could be maintained long-term [15]. In the current study, we describe the results of long-term (more than three years) *in vitro* growth of these GSCs and their detailed characterization. To ensure that GSCs are available for use when required, the cells were periodically cryopreserved and resuscitated during long-term culture and tested for their capacity to form new nonadherent tumor spheres upon retrieval. We also compared the biological characteristics of GSCs derived from the primary and recurrent tumors in a patient with malignancy progression.

## Methods

### Clinical Information

**Figure 1:**
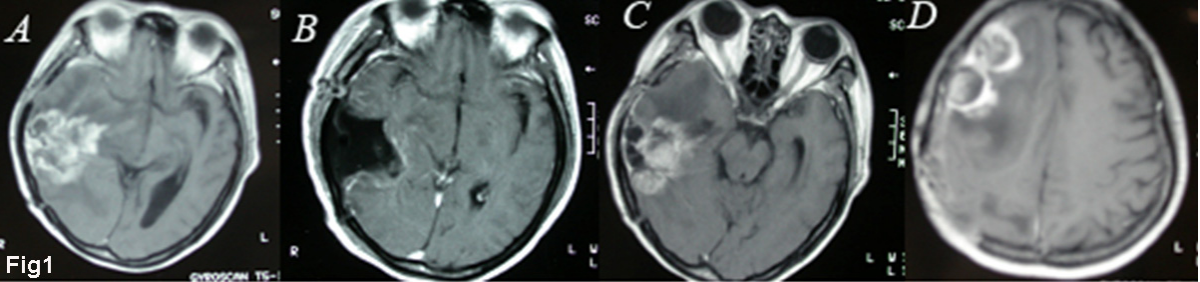
**MRI images of a 52-year-old female patient who underwent two operations with an interval of 6 months because of the rapid relapse of her tumor.** A, The primary tumor located in the right temporal lobe and pathologically diagnosed as mixed glioma composed of anaplastic ependymoma and astrocytoma (WHO grade III). B, Post-operation image showing nearly total resection of the tumor. C, D, Relapse occurring 6 months later in situ and the ipsilateral frontal lobe; the recurrent lesion had transformed and progressed into glioblastoma multiforme (WHO grade IV).

Our studies were approved by the Medical Review Board of Suzhou University Medical School. Signed informed consent was obtained from the patients or their legal guardians prior to sample acquisition. Fresh surgical specimens were obtained from a 52-year-old female patient who had undergone two operations with an interval of 6 months because of the rapid re-growth of tumor mass in the right temporal lobe. The patient had received one dose of radiotherapy (45 Gy) one month after the initial surgery, then three doses of chemotherapy (VM26 0.1 ivgtt × 3 days followed by MeCCNU 200 mg PO once) at one-month intervals. In recurrence, a new solitary focus emerged in the ipisilateral frontal lobe in addition to tumor re-growth in situ. The primary lesion was pathologically diagnosed as mixed glioma comprising anaplastic ependymoma and astrocytoma (WHO grade III), while the recurrent lesion had transformed and progressed into glioblastoma multiforme (WHO grade IV) (Fig. 1) despite the continuous post-surgical radiotherapy and chemotherapy.

### Isolation and culture of glioma stem cells

Tumor tissues were washed and minced with fine scissors into small fragments. Single tumor cells and small clumps (3–5 cells per clump) were collected with a 35 μm cell strainer, then resuspended in DMEM-F-12 culture medium (Gibco-Invitrogen) to achieve a final concentration of 1 × 10^8 ^live cells per ml as assessed by Trypan blue staining [16]. Tumor spheres were cultured as described previously with some modifications [15].

### Flow cytometry and FACS of CD133-positive cells

A single cell-suspension from the tumor spheres was centrifuged, and magnetic cell separation and fluorescence-activated cell sorting were performed as follows. The cells were dissociated and resuspended in PBS. For magnetic labeling, CD133/1 Micro Beads were used (Miltenyi Biotech). Positive magnetic cell separation (MACS) was carried out using several MACS columns in series according to the manufacturer's instructions (Miltenyi Biotec). Cells were labeled with phycoerythin (PE)-conjugated monoclonal antibodies against human CD133 (CD133/2-PE, Miltenyi Biotec) or isotype control antibody (IgG2b(mouse)-PE, Caltag Laboratories) and analyzed using a BD FACS Calibur. The isolated CD133+ cells were suspended in defined stem cell growth medium containing DMEM-F-12, N_2 _supplement (Gibco), penicillin G, streptomycin sulfate, recombinant human FGF-2 (20 ng/ml; R&D System) and recombinant human EGF (20 ng/ml; R&D Systems), and were plated at a density of 2 × 10^6 ^live cells per 75 cm^2 ^on an uncoated plastic flask.

Aliquots of CD133+ tumor cells obtained from MACS were further flow-sorted by FACS. The cells were labeled with CD133/2-PE at 4°C for 10 min following the manufacturer's instructions, then washed and resuspended in stem cell growth medium; negative controls were performed with IgG2b(mouse)-PE antibody as recommended by the manufacturer. The cells were then flow-sorted and dead cells were excluded by propidium iodide (PI) staining.

### Subsphere formation

Tumor spheres of different passages were dissociated into single cells and plated at a density of one cell per well. Half the volume of each culture medium (50 μl) was changed every three days. The formation of tumor spheres was observed under a phase-contrast microscope.

### Proliferation

For proliferation assays, cells were plated on 96-well plates and cultured in 200 μl stem cell growth medium/well at a density of 2000 cells/ml. From day 0 to day 10 after plating, viable cells were quantified using the 3-(4,5-dimethylthiazol-2-yl)-2, 5-diphenyltetrazolium bromide-based Colorimetric Assay Cell Proliferation kit (Roche) according to the manufacturer's instructions.

### Tumor spheres cultured alternately in serum-based medium and serum-free stem cell growth medium

Nonadherent tumor spheres were seeded into DMEM-F12 medium supplemented with 10% fetal bovine serum (FBS, Hyclone). After the spheres had attached to the bottom of the flask and grown into cell monolayers for two weeks, the cells were washed with PBS to remove fetal bovine serum and transferred to defined stem cell growth medium. These procedures were repeated and morphological changes in the tumor cells were observed under a phase-contrast microscope.

### Immunofluoresence staining to detect the expression of differentiation markers

Isolated CD133+ cells were cultured in the aforementioned stem cell growth medium to allow tumor spheres to form. To determine the capacity of CD133+ cells for multi-lineage differentiation, the tumor spheres were transferred to poly-D-lysine coated chamber slides and cultured in DMEM-F12 supplemented with 10% FBS. For immunofluorescence staining at different times during differentiation, namely days 0 (cultured for 4 h in serum-based medium), 3, 7 and 10, cells grown on the slides were fixed with 4% paraformaldehyde and permeabilized with 0.1% Triton X-100. The slides were then probed with mouse antibodies against human CD133, nestin (BD Bioscience), GFAP (Santa Cruz) and β-Tubulin-III (BD Bioscience). The secondary antibodies were either FITC- or Texas Red-conjugated anti-mouse IgG (Vector Laboratories). The cells were counterstained with 4',6'-diamidino-2-phenylindole (DAPI; Vector Labs). Expression and/or coexpression of the aforementioned cell surface markers during differentiation was detected with a laser scanning confocal microscope (Carl Zeiss), and images were captured on a color CCD at specific magnifications.

### Tumorigenicity of GSCs

Tumor cells from both SU-1 and SU-2 spheres were collected and suspended in 2 μl PBS, then injected intracranially into the right caudate nucleus of athymic (NCR nu/nu) mice. All procedures were conducted in accordance with Chinese laws governing animal care. Briefly, under the guidance of a stereotactic system, 2 μl cell suspension (1 × 10^8 ^cells/ml) in PBS were delivered into the right caudate nucleus (0.2 μl/min) by injection through a glass electrode connected to a Hamilton syringe. Mice were sacrificed when they became moribund or showed signs of obvious neurological deficit. Tumor samples were snap-frozen and frozen sections (10 μm) were stained with HE following standard protocols.

### Transmission electron microscopy

A single cell suspension from the tumor spheres was centrifuged, and magnetic cell separation and fluorescence-activated cell sorting were performed to collect CD133+ GSCs. Positive cells were resuspended in PBS and the suspension was fixed in 4% buffered glutaraldehyde, dehydrated through a graded ethanol series, embedded in Epon and cut into thin sections. The samples were imaged by a transmission electron microscope.

### Array-based comparative genomic hybridization (array-CGH)

Genome-wide array comparative genomic hybridization was performed to determine changes in the copy number of genomic DNA from SU-1 and SU-2 GSCs. The array used in this study consisted of 2632 human BACs (Spectral Genomics, Houston, TX) spaced at approximately 1 megabase (Mb) intervals across the whole genome. The experiments were performed according to the manufacturer's protocol, as described previously [17]. Briefly, the arrays were pre-hybridized with human Cot-I DNA (Gibco Invitrogen, Carlsbad, CA) and salmon testis DNA to block the repetitive sequences on BACs. One microgram of normal DNA (reference) or tumor DNA (test) was labeled with cy5-dUTP and cy3-dUTP by random priming. To avoid dye bias, we performed dye swap experiments for each sample. The probe mixture was dissolved in hybridization mixture, denatured, cooled and mounted with a 22 × 60 mm coverslip. Hybridizations were performed in sealed chambers for 16–20 h at 60°C. After post-hybridization washes, the arrays were scanned into two 16-bit TIFF image files using a GenePix 4000A two-color fluorescent scanner and quantified using GenePix software (Axon Instruments, Union City, CA). Data were analyzed using SpectralWare 2.2.23 (Spectral Genomics, Houston, TX).

### Quantitative real-time PCR

Real-time PCR was performed using SYBR green reagent and the ABI PRISM 7000 Sequence Detection System (PE Applied Biosystems) according to the manufacturer's instructions. *β-actin *transcripts were quantified in all samples as an internal control for the amount and quality of cDNA. Detailed information about primer sequence and product size is available upon request. The primer sets were all optimized to generate a single specific band only from cDNA on argarose gels. Melting-curve analysis was performed for all the reactions to control for the specificity of amplifications. The results of real-time PCR were analyzed by the ΔΔCt method and presented as the ratio between the selected genes and *β-actin *transcripts. The selected gene/*β-actin *ratio was then normalized to the mean ratio of the selected genes in normal peripheral blood (for DNA) or in cultured normal human astrocytes (Cambrex, East Rutherford, NJ) (for mRNA) to calculate the Tumor/Normal ratio. All experiments were performed in triplicate.

## Results

### Growth and differentiation of GSCs maintained long-term *in vitro*

**Figure 2:**
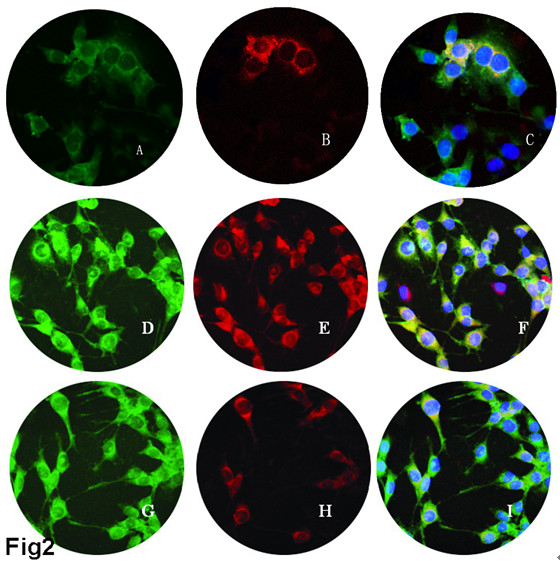
**Expression of cell surface markers of tumor spheres before and after differentiation (×400).** A-C, tumor spheres before differentiation. D-I, one week after FBS-induced differentiation. A, D, G, immunofluorescence staining for nestin (green). B, immunofluorescence staining for CD133 (red). E, immunofluorescence staining for GFAP (red). H, immunofluorescence staining for β-tubulin-III (red). C, F, I, confocol microscopy showed co-expression of nestin with CD133, GFAP and β-tubulin-III, respectively.

Glioma stem/progenitor (GSPC) cell lines, named SU-1 for the primary tumor and SU-2 for the recurrent tumor associated with malignancy progression, were maintained *in vitro* for more than 44 months and 38 months, respectively. Both lines contained an obvious CD133+ subpopulation that displayed neurosphere-like tumor spheres, nonadherent growth and asymmetrical cell divisions yielding cells that expressed markers of neuron and glia differentiation. The expression and coexpression of differentiation-related surface makers were examined by laser scanning confocal microscopy. Within a single sphere grown from one CD133+ GSC, the cells expressed markers of different neural lineages at constant proportions; most of the tumor cells did not differentiate terminally and often co-expressed differentiation markers with the stem/progenitor cell marker nestin. Over 90% of the cells in both SU-1 and SU-2 tumor spheres were nestin positive (Fig. 2A) and about 5–10% stained positively for CD133 (Fig. 2B). When the tumor spheres became adherent monolayers, one week after FBS-induced differentiation without growth factor supplementation *in vitro*, about 78–83% of the cells were still nestin positive (Fig. 2D, 2G). However, about 17–22% of these nestin+ cells were also positive for such differentiation markers as GFAP for astroglial cells (Fig. 2E) and/or β-tubulin-III for neurons (Fig. 2H), confirming that they retained multi-lineage differentiation capabilities. Therefore, GSCs cannot reach the stage of complete terminal differentiation under conditions that would induce terminal differentiation in NSCs. *In vitro* data suggest that the differentiation of most GSCs is blocked at the neural progenitor stage, as indicated by the expression of nestin, or at the early stage of tumor progenitor cell differentiation, as indicated by the partial co-expression of nestin and glial/neural differentiation markers.

**Figure 3:**
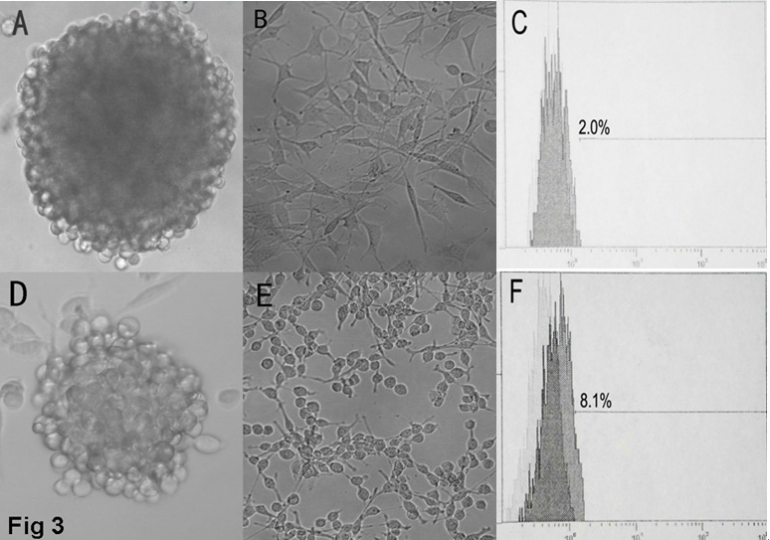
**Culture of SU-1 and SU-2 cells *in vitro*.** A, tumor sphere of SU-1 (250×). B, differentiated SU-1 (100×). C, percentage of CD133+ SU-1 cells detected by FCM. D, tumor sphere of SU-2 (250×). E, differentiated SU-2 cells (250×). F, percentage of CD133+ SU-2 cells detected by FCM.

Nonadherent tumor spheres were dissociated and magnetically sorted into CD133+ and CD133- cells. Upon replating at one cell per well, tumor spheres formed from single CD133+ cells, often reaching a size of 30–40 cells in approximately two weeks. Only a small proportion (4.5% for SU-1 and 12.8% for SU-2) of the CD133+ tumor cells formed spheres, but no *in vitro* sphere formation was observed with CD133- cells. Sequential minimal dilution assays for three passages confirmed that the CD133+ single cell-derived tumor spheres had the potential to grow indefinitely. The proportion of sphere-forming cells remained stable throughout the course of culture, indicating asymmetrical cell divisions. Periodically, the tumor sphere cells were cryopreserved and resuscitated; the resuscitation rate was about 75%. However, SU-1 and SU-2 showed some morphological differences *in vitro*. Briefly, when cultivated in defined stem cell growth medium (FBS free), the SU-1 tumor spheres were more compact while the SU-2 ones seemed a little looser and were not evenly round (Fig. 3A, 3D). When cultured in medium containing 10% FBS, both SU-1 and SU-2 cells changed morphologically from rolling spheres to adherent monolayers. Most of the differentiated SU-1 cells were star- or spindle-shaped (Fig 3B), but most of the differentiated SU-2 cells were round or oval (Fig 3E). Fluorescence-activated cell sorting confirmed the presence of CD133+ cells. The relative content of CD133+ cells was markedly higher in the recurrent cell line SU-2 (8.1%, Fig 3F) than in the primary tumor cell line SU-1 (2.0%, Fig 3C). Non-specific staining was 1.3% for SU-1 and 0.6% for SU-2.

### Intracranial xenografts derived from SU-2 are more aggressive than those from SU-1

**Figure 4:**
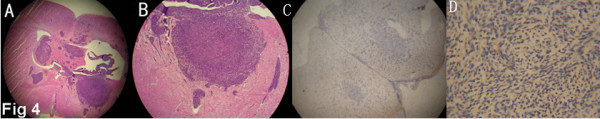
**Pathological examination of intracranial tumor mass in NC nude mice.** A, SU-1 cells produced relatively well-delimited tumor masses (HE × 20). B, local amplification of A, showing nodular tumor formation (HE × 100). C, SU-2 cells behaved more aggressively (HE × 20). D, SU-2 cells infiltrated remoter regions and invaded the whole brain evenly and densely (HE × 200).

When inoculated intracranially into athymic (NCR nu/nu) mice, both SU-1 and SU-2 reproducibly established tumors (5/5 for both SU-1 and SU-2). The average median survival time was 32 days for SU-1 and 27 days for SU-2. Pathologically, SU-1 cells gave rise to less invasive tumor masses; most of the tumor was restricted to the ipsilateral hemisphere of the injection site, and nodular tumor formation was observed (Fig. 4A, 4B). In contrast, tumors derived from SU-2 cells showed diffuse infiltrating growth patterns; they were distinctive in that they were widely disseminated, invaded the ipsilateral hemisphere, migrated to the cortex on the contralateral side and infiltrated the subarachnoid space of the longitudinal fissure, recapitulating the original recurrent lesion (Fig. 4C). Moreover, most tumor cells were primitive round or oval, distributed evenly and densely without forming obvious tumor masses, morphologically resembling gliomatosis cerebri (Fig. 4D), indicating that the latter may be a stem cell disease. This possibility deserves further investigation.

### Transmission electron microscopy showed that GSCs from both SU-1 and SU-2 lacked autophagosomes

**Figure 5:**
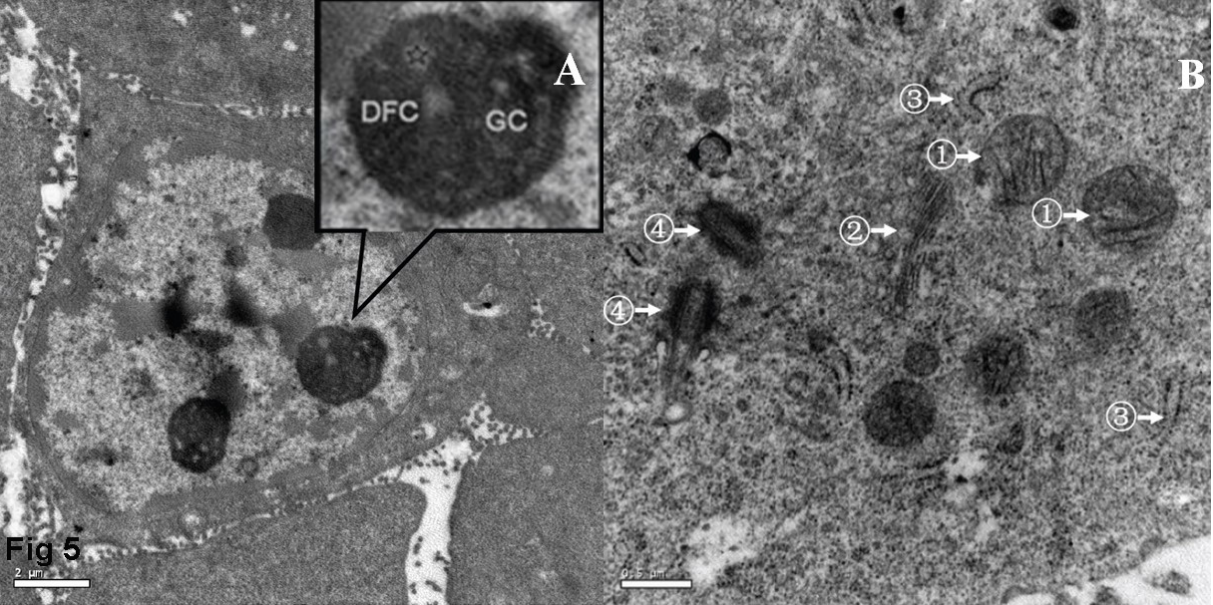
**GSCs observed under transmission electron microscopy (TEM) showed a lack of autophagosomes.** A, nuclei and chromatin, showing a high nucleus: cytoplasm ratio, large oval nucleus and nucleoli (the asterisk indicates the FC). B, mitochondria (arrow ①), Golgi apparatus (arrow②), rough endoplasmic reticulum (arrow ③), a couple of centrioles (arrow ④).

Examined by transmission electron microscopy, the nuclei of GSCs from SU-1 and SU-2 were large and regular, circular or oval in shape, with typical inner and outer nuclear membranes, nucleopores, perinuclear space and other structures. Euchromatin was far more abundant than heterochromatin. Two or more nucleoli were often observed with clear fibrillar centers (FC), dense fibrillar components (DFC) and granular components (GC). In the cytoplasm, ribosomes and mitochondria were abundant while the Golgi apparatus and endoplasmic reticulum were relatively less developed; lysosomes (including autophagosomes) were rare in comparison with NSCs. Taken together, these ultrastructural features implied that the GSCs were at a primitive stage of differentiation with low autophagic activity (Fig. 5).

### Clones with altered DNA copy number identified by array-based CGH

**Figure 6:**
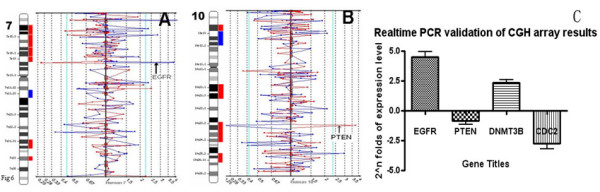
**Copy number changes in genomic DNA from GSCs of both SU-1 and SU-2 lines were identified by the CGH array, the ideograms show the gains (blue) and losses (red) of DNA copy numbers.** Representative ratio plots of chromosomes 7, with amplification of oncogene *EGFR *(A), and 10, with deletion of tumor suppressor *PTEN *(B); Real-time quantitative PCR validated some of array-based CGH results (C).

Among the 2621 clones spotted on the array, 2457 were mapped to autosomes. Changes in the DNA copy number were found in 160 clones of GSCs from SU-1 and 182 clones from SU-2. With increased resolution of the BAC array, regions with changes in DNA copy number spanning more than 1 Mb could be detected in an average of two clones. The clustered changes were defined as regions with at least two consecutive adjacent clones within 1 Mb on the linear map of the human genome sequence. A total of 7 clustered regions on chromosomes 1p, 2p, 2q, 4q, 9p, 14q and 19q were identified in GSCs of both SU-1 and SU-2 (Additional file 1). Subsequent searching of the human genome draft revealed several prominent genes in these regions that have often been associated with human cancer, and these were also found to be altered (Additional file 1). Small gains or losses restricted to single BAC clones were also detected. Most of the altered clones were detected in GSCs from both SU-1 and SU-2. Among the isolated BAC clones in which the tumor/normal ratio exceeded 1.3 (gain) or was less than 0.75 (loss), a clone on 7p12 containing the gene for epidermal growth factor receptor (*EGFR*) was amplified (Figure 6A). The clone RP11-129G17 on 10q23 had the lowest tumor/normal ratio. Since the tumor suppressor gene *PTEN *is mapped exactly to the region covered by RP11-129G17, this finding suggests that *PTEN *was deleted in GSCs of both the SU-1 and SU-2 lines, as has frequently been reported in gliomas (Figure 6B). These genetic changes included deletion of *p18INK4 *and *Rb *as well as the tumor suppressor *PTEN*, and amplification of the oncogenes *CDK7 *and *EGFR*. We also found some genetic changes that were exclusive to either SU-1 or SU-2. The most intriguing of these was the amplification of *MTA 1 *in SU-2 (metastasis associated 1), a gene previously found to be closely related to the recurrence and metastasis of breast cancer, osteosarcoma and other malignances (Additional file 2).

### Real-time PCR validated the results of the array-based CGH

To validate the array-based CGH findings, quantitative real-time PCR was performed to examine the expression of genes encoding *EGFR*, *PTEN*, *CDC2 *and *DNMT3B*. Consistent with the array-based CGH results, real-time PCR showed increased expression of *EGFR *and *DNMT3B *and down-regulation of *PTEN *and *CDC2 *in GSCs compared to normal human NSCs (Figure 6C).

## Discussion

In the past few years, stem cell-like tumor precursors have been identified in gliomas. They have been consecutively termed glioma stem cells, brain tumor stem cells or brain tumor initiating cells. They are characterized by self-renewal, limitless proliferation, tumor initiation, multi-differentiation and expression of stem cell surface markers such as CD133 and nestin. However, long-term stable maintenance of GSCs, which will offer much more convenient opportunities for attaining full and accurate understanding of the biological features of this special tumor cell type, has been achieved by only a few groups and does not suffice to meet research requirements [18,19]. No pure CD133+ glioma stem cell line has so far been available; proliferation and differentiation of these tumor stem cells *in vitro* cannot be stopped completely even in a culture medium favoring stem cell growth. The percentage of CD133+ cells in such lines has varied widely. Accordingly, there are no unanimously agreed criteria for establishing a GSC line. Successful cell lines from other tumors suggest that establishment of a GSC line should meet the following criteria. First, GSCs can be cultured long-term *in vitro* while maintaining relatively stable stem cell properties. Secondly, even after long-term maintenance, the GSCs should recapitulate their parent or original tumor. In the current study, SU-1 and SU-2, respectively originating from primary and recurrent gliomas with malignancy progression in the same patient, have been maintained *in vitro* for more than three years while retaining their tumor stem cell properties. Though the percentage of CD133+ cells was not high (less than 10%), nestin+ cells were the dominant subgroup (> 90%). Thus, the two newly established cell lines SU-1 and SU-2 could be regarded as glioma stem/progenitor lines. Cryopreservation and resuscitation were successful during long-term serial passages *in vitro*. We also noticed differences in configuration between the tumor spheres derived from SU-1 and SU-2. When cultured in defined stem cell growth medium (FBS free), the SU-1 spheres were more compact than those of SU-2, and the percentage of CD133+ cells was lower. When cultured in serum-based medium, SU-2 seemed more resistant to FBS-induced differentiation and remained more morphologically primitive than SU-1. In vivo, direct orthotopic transplantation of SU-1 and SU-2 cells developed into xenografts in immune-deficient mouse cerebrum, but the tumors derived from SU-2 cells were more aggressive than those from SU-1. These data imply that malignancy progression could also occur in tumor stem cells. Taken together, these results suggest that SU-1 and SU-2 could provisionally be regarded as permanent glioma stem/progrnitor cell lines and further utilized as reliable resources for basic research and clinical trials concerning GSCs.

The study of GSCs is actually an extension of that of NSCs, since not only the concepts but also the methods employed are derived from those used for NSCs. The finding that 10^2 ^CD133+ tumor cells could produce tumor mass in NOD-SCID mice, while up to 10^5 ^CD133- tumor cells could not, proved that the former were brain tumor initiating cells and the latter were not [8]. So it seemed reasonable to suppose that CD133+ tumor stem cells could proliferate and differentiate into CD133- cells, which could further differentiate into common tumor cells approaching terminal differentiation, as NSCs do. However, Beier's studies revealed that four of 15 cell lines derived from primary glioblastomas grew adherently *in vitro* and were driven by CD133- tumor cells that fulfilled stem cell criteria. Both CD133+ and CD133- subtypes of GSCs were similarly tumorigenic in nude mice in vivo [20], indicating that CD133 expression is not sufficient to identify GSCs; more effort is needed to identify a specific GSC marker. At present, though this functional criterion for GSCs is sophisticated and inconvenient to apply, it is reliable and should not be neglected unless and until a specific marker for GSCs is found.

GSCs do not differentiate terminally under conditions that would induce terminal differentiation in NSCs. Not only was differentiation retarded, but retro-differentiation was also observed *in vitro*. Our data showed that soon after treatment with differentiation-inducing agents such as FBS and valproate (VPA), nonadherent tumor spheres dissociated and scattered into adherent spindle-shaped monolayer cells. Most of these were still highly positive for nestin (a marker for neural stem/progenitor cells), while a few cells appeared that were doubly positive for nestin and either GFAP (marker for astrocytes) or β-tubulin III (marker for neurons). Markers of both mature and stem/progenitor cells are very rarely co-expressed during NSC differentiation, but it is common in GSCs [15]. We also observed a "down-up" trend in the percentages of CD133+ cells in SU-1 and SU-2 during a relatively long differentiation-inducing process *in vitro*; that is, the percentage of CD133+ cells decreased at first, then remained low for a time and finally increased a little, suggesting that partially differentiated CD133+ cells (loss of CD133 expression) retro-differentiated into CD133+ GSCs under certain circumstances, which made the GSCs involved in tumor remodeling more sophisticated. There was a concomitant "up-down" trend in the levels of the neural differentiation markers GFAP and β-tubulin-III. These phenomena were more obvious in SU-2 [15]. Thus, it is easy to infer that GSCs were generally similar to NSCs but showed important differences. Under conditions in which differentiation would be induced in NSCs, GSCs showed an intrinsic potential to maintain their undifferentiated state or to resist differentiation and even tended to retro-differentiate under certain circumstances. Once differentiation was initiated in NSCs, they were transformed step by step into various kinds of mature neural cells.

Amplification of the oncogene *EGFR *and deletion of the tumor suppressor *PTEN *have been identified as the critical genetic changes in the tumorigenesis of human GBMs or other types of glioma. However, few existing glioma cell lines harbor these genetic abnormalities [21-32]. The fact that GSCs of both the SU-1 and SU-2 lines faithfully preserved the *EGFR *amplification and *PTEN *loss greatly enhances their utility in biological and preclinical studies of human gliomas. Recent studies have shown a close correlation between *PTEN *loss and low autophagic activiy [33]. We also found that *PTEN *loss and absence of autophagy were concurrent in both SU-1 and SU-2, and this may suggest potential targets for future molecular intervention. More intriguingly, we discovered amplification of *MTA1 *in SU-2 but not in SU-1. *MTA1 *is closely associated with various malignancies and its up-regulation always indicates tumor recurrence and metastasis [34-39]. However, the significance of *MTA1 *in the malignancy progression of gliomas has rarely been considered. In the current study, the particular amplification of *MTA1 *in GSCs derived from the recurrent tumor makes it reasonable to conjecture that *MTA1 *activation may contribute to both the aggression of GSCs and the malignancy progression of gliomas.

## Conclusion

In summary, we successfully established two glioma stem/progenitor cell lines from primary and recurrent tumors with malignancy progression obtained from the same patient. We discovered that GSCs in the recurrent tumor with malignancy progression were more aggressive than in the primary tumor, which suggests that tumor progression may be initiated early in tumor stem cells. We also demonstrated that direct isolation and long term maintenance of GSCs from freshly resected glioma tissues is a feasible approach for future biological studies of cancer stem cells and pre-clinical testing of novel therapeutic agents.

## Competing interests

The authors declare that they have no competing interests.

## Authors' contributions

QH participated in the study design, carried out most of the experiments and drafted the manuscript. QBZ carried out the immunoassays and participated in the critical revision of the manuscript. JD conceived of the study, participated in its design and coordination and participated in manuscript preparation. YYW participated in clinical data collection and critically revised the manuscript. YTS carried out the stem cell culture. YDZ performed the ultramicroscopy of the stem cells. YDZ carried out the flow cytometry assay. YD performed the animal experiments. ADW carried out the molecular genetic studies and the critical revision of the manuscript. QL participated in the coordination of the study and the critical revision of the manuscript. All authors read and approved the final manuscript.

## Consent

Written informed consent was obtained from the patient for publication of this article and accompanying radiographic images.

## Pre-publication history

The pre-publication history for this paper can be accessed here:



## Supplementary Material

Additional file 1 Summary of clustered DNA copy number changes in GSCs detected by CGH array Click here for file

Additional file 2 Click here for file
