# Supplementary material for: Glioma stem cells are more aggressive in recurrent tumors with malignant progression than in the primary tumor, and both can be maintained long-term in vitro
Source: BMC Cancer. 2008 Oct 22;8:304. doi: 10.1186/1471-2407-8-304 (PMC2584338; doi:10.1186/1471-2407-8-304)
Supplement: Additional file 1 — Summary of clustered DNA copy number changes in GSCs detected by CGH array [file 1471-2407-8-304-S1.doc]

| Table 1  Summary of clustered DNA copy number changes in GSCs detected by CGH array | | | | | | | |
| --- | --- | --- | --- | --- | --- | --- | --- |
| **Chromosome Cytoband** | **Linear range (Mb)*** | **p-Terminal clone ID** | **q-Terminal Clone ID** | **Total clones** | **DNA Change** | **Gene Span** | Genes associated with Glioma**/**Tumor |
| 1p32.1-1p33 | 51.3-52.1 | RP11-253A20 | RP4-814E15 | 2 | LOSS | *FAF1-FLJ14936* | *CDKN2C,EPS15,RAB3B* |
| 2p12 | 81.8-82.8 | RP11-89C12 | RP11-345F13 | 2 | LOSS | *LOC442021* | *LOC442021* |
| 2q34 | 213.5-214.6 | RP11-560C24 | RP11-44J16 | 2 | LOSS | *ERBB4-PF20* | *ERBB4* |
| 4q25-4q27 | 112.7-112.9 | RP11-18D18 | RP11-89D13 | 2 | LOSS | *LOC391686-LOC402184* |  |
| 9p21-9p22.1 | 18.6-18.6 | RP11-399M15 | RP11-81B11 | 2 | LOSS | *ADAMTSL1-FAM29A* |  |
| 14q12-14q13.1 | 31.1-31.8 | RP11-54H22 | RP11-557O15 | 2 | LOSS | *AP4S1-MTCO1P2* |  |
| 19q13.3-19q13.4 | 57.2-57.9 | RP11-79A3 | RP11-79I16 | 2 | GAIN | *SIGLEC6-FLJ32214* |  |

* Linear range shows the location of the BACs relative to their megabase position along the length of the individual chromosome from the human genome sequence draft.
