# Supplementary material for: Glioma stem cells are more aggressive in recurrent tumors with malignant progression than in the primary tumor, and both can be maintained long-term in vitro
Source: BMC Cancer. 2008 Oct 22;8:304. doi: 10.1186/1471-2407-8-304 (PMC2584338; doi:10.1186/1471-2407-8-304)
Supplement: Additional file 2 [file 1471-2407-8-304-S2.doc]

Table 2. Genetic changes exclusively found in GSCs of SU-1 or SU-2 by array-based CGH

| **GSCs** | **Clone ID** | **Chromosome cytoband** | **Linear map (Mb)** | **DNA Change** | **Genes associated with Glioma/Cancer** |
| --- | --- | --- | --- | --- | --- |
| SU-1 | C197-2 | 17p13.3 | 2.2 | L | OVCA2 |
|  | CTB-41I5 | 17p13-17p13 | 8.8 | L | CAS7 |
|  | RP11-90G3 | 12q15-12q15 | 70.6 | G | RAB21 |
| SU-2 | RP11-89F1 | 5q32-5q32 | 147.5 | L | LATS1 |
|  | RP5-1099D15 | 20p12.2 | 10.5 | L | FAT1P1 |
|  | CTC-200D12 | 14q32.33 | 105.1 | G | MTA1 |
|  | RP1-148L21 | 1q21.2-1q22 | 150.2 | G | RAB13 |
|  | RP11-12M5 | 1q24.1-1q25.3 | 176.3 | G | RASAL2 |
|  | RP11-7020 | 11p15.2-11p15.2 | 15 | G | RRAS2 |
|  | RP11-391H12 | 13q34 | 112.9 | G | RASA3 |

G: Gain; L: Loss
